# Modeling and simulation of flotation reagents system in anionic reverse iron oxide flotation at different temperatures

**DOI:** 10.1038/s41598-023-35187-4

**Published:** 2023-05-19

**Authors:** Ying Hou, Ahmed Sobhy

**Affiliations:** 1 School of Mining Engineering, University of Science and Technology Liaoning, Anshan, 114051 China; 2 School of Resources and Environmental Engineering, Shandong University of Technology, Zibo, 255049 China; 3 Minerals Technology Department, Central Metallurgical Research and Development Institute, Helwan, 11421 Cairo Egypt

**Keywords:** Engineering, Mathematics and computing, Mineralogy

## Abstract

Removal of quartz from iron ore was accomplished industrially via an anionic reverse flotation technique. However, in that kind of flotation, the interaction of the flotation reagents with the components of the feed sample makes the flotation a complicated system. Thus, the selection and optimization of regent dosages at various temperatures were performed using a uniform experimental design to estimate the optimum separation efficiency. Besides, the produced data as well as the reagent system were mathematically modeled at different flotation temperatures, and the graphical user interface GUI of MATLAB was conducted. The advantage of this procedure is that the user interface displayed in real-time can be conducted by adjusting the temperature at different values to automatically control the reagent system, besides predicting the concentrate yield, total iron grade, and total iron recovery.

## Introduction

Iron ores are the primary source of iron which is essential for the world’s iron and steel industries and serve as the backbone for the country’s basic infrastructure improvement. The iron ores consist of iron oxides mainly of magnetite (Fe_3_O_4_) and hematite (Fe_2_O_3_). However, the average grade of these ores in China is less than the cut-off grade of 45%^1^. Thus, upgrading process development was required for increasing the grade of the ores to meet the demand of the iron and steel industries. The beneficiation was studied extensively^2–4^ which consists of several stages, and anionic reverse flotation was the common route in the processing plants to produce the final concentrate^5–9^. On the other hand, direct flotation as an alternative route led to a partial flotation of quartz and in consequence reduced the grade of the final concentrate^10^. However, the anionic reverse flotation is established at a pH value of 11.5 to maximize the repulsive electrostatic force between negatively charged iron oxide (valuable) and the quartz (gangue) particles. Also, a depressant such as corn starch is utilized to inhibit the floatability of iron oxide particles besides aggregating fine iron oxide particles to not be transported to the froth zone^11^. In addition, an activator such as calcium oxide is used to activate selectively the quartz by changing its surface charge into positive. Then an anionic collector like commercial TD-II is adsorbed on the positively charged quartz to increase its hydrophobicity and in consequence its floatability^12–14^. Thus, the appropriate reagent system in anionic reverse flotation is very critical and complicated especially when dealing with low-grade iron ores as a result of the interaction of the different flotation reagents with the diverse components of the feed slurry. Thus, the selection and optimization of the reagent system are vital investigation stage which needs a lot of effort and is time-consuming, thus a uniform experimental design has been conducted by the authors^1^. Some of the advantages of uniform test design are a very quick, highly efficient, economical experimental design method that reduces test times, shortens test cycles, and is capable of quickly finding multi-factor optimization schemes^15,16^.

The anionic reverse flotation of iron oxide is usually carried out with automatic slurry temperature control at 35 °C^12,14^ or 30 °C^1^. In this paper, the flotation experiments of the iron oxide feed were carried out using the uniform test design method at different flotation temperatures from 20 °C up to 45 °C to investigate and optimize the influences of the flotation reagents as a function of flotation temperature.

In this type of research, various diagrams can be quickly generated using a MATLAB-based graphical user interface GUI. It is one of the fast and most common software used for data analysis and visualization, algorithm calculation, and numerical design^17^.

## Methods

### Materials

The representative experimental samples were collected from the flotation circuit feed of the Anqian processing plant located in Anshan, Liaoning. The industrial plant runs a flotation circuit with samples having 90%, 50%, and 10% finer than 104 μm, 35 μm, and 6 μm respectively; and total average iron content of about 48%. The complete sample characterizations were illustrated in previous work using X-ray diffraction (XRD), X-ray fluorescence, and Mineral Liberation Analyzer (MLA)^1,12,14^. The major minerals are iron oxide (hematite/magnetite) and quartz with other impurities such as pyrite and apatite^1,12,14^.

The flotation circuit is conducted based on the typical plant conditions using the plant chemical reagents including starch as iron oxide depressant, lime (CaO) as quartz activator, and commercial anionic TD-II as quartz collector. Besides, analytical grade NaOH and HCL (15% w/w) were used as pH modifiers.

### Flotation process

**Figure 1:**
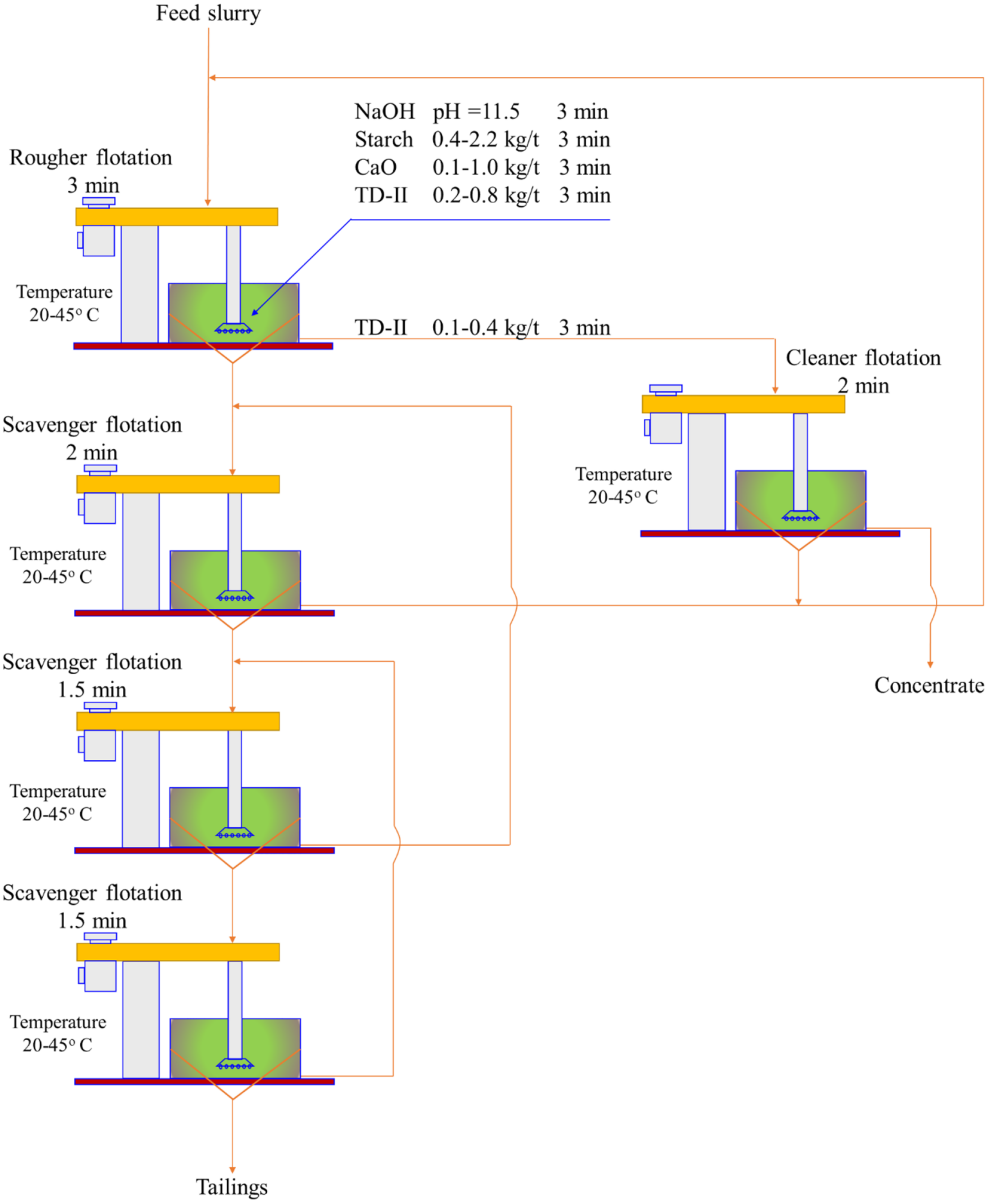
Flowsheet of reverse anionic flotation of iron oxide.

The studies were conducted using the plant flowsheet shown in Fig. 1 but at different flotation temperatures from 20 °C up to 45 °C. The closed circuit consists of roughing, cleaning, and three scavenging flotation stages at 11.5 pH value^1^. At the roughing stage, depressant, activator, and collector are added, while a third of the collector is added in the cleaning stage. The tailing of the cleaner is combined with the concentrate of the first scavenger and sent back to the rougher feed, the concentrate of the second scavenger is recycled back to the first scavenger feed, and the concentrate of the third scavenger is sent back to the second scavenger feed (Fig. 1). The products of the flotation circuit were dewatered, dried, homogenized, weighed, and the total iron grades were analyzed.

### Uniform test design

For the flotation circuit of iron oxide, a uniform test design was conducted to estimate the optimum flotation reagent systems at different flotation temperatures from 20 °C up to 45 °C. The detailed uniform design optimization methodology was given in previous work^1^. Uniform test design is a computer simulation accomplished in many industrial applications to produce a model presenting the real process performance. Thus, uniform test design U_10_(10^3^) experiments of 10-run for 10-level of 3 factors for each flotation temperature was done in a randomized order to obtain the optimum flotation reagent system giving the best separation efficiency. The dosage range of each factor was identified through the preliminary tests to be 0.4–2.2, 0.1–1.0, and 0.3–1.2 kg/t for the depressant, activator, and collector respectively. The interactions between the factors in terms of separation efficiency were elucidated using response surface modeling.

### Modeling and simulation

**Figure 2:**
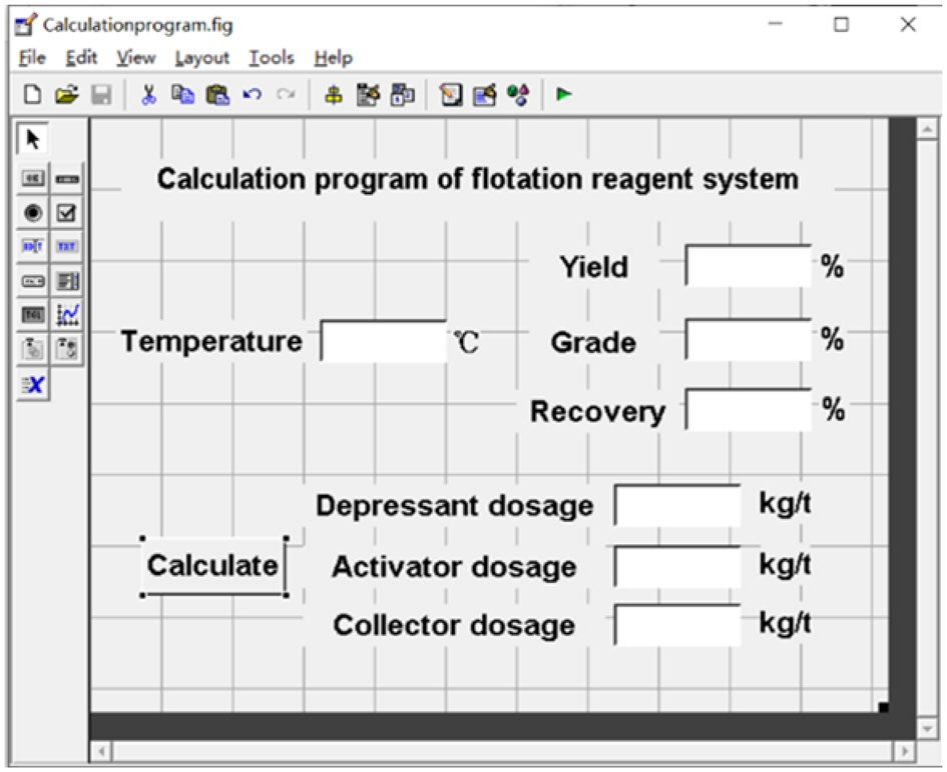
The operation and contents interface of the MATLAB calculation program of the flotation reagent system.

GUI of MATLAB was conducted to simulate the optimum flotation reagent system at various flotation temperatures based on the estimated optimum data using a uniform test design. The interface content design of the flotation reagent system calculation program is shown in Fig. 2.

The layout shows the flotation temperature as an input value, while the values of depressant dosage, activator dosage, collector dosage, concentrate yield, grade, and recovery are output after clicking the calculation button which is the execution command button.

The calculation procedure in Code View was as follows:

- Function pushbutton3_Callback(hObject, eventdata, handles).
- a = get(handles.edit1,'string');
- % Obtain the flotation temperature:
- dianfen = 1000 × (48.1 × (str2double(a)) + 79.88)/((str2double(a))^2–57.17*(str2double(a)) + 1798);
- % Calculate the dosage of depressant:
- yanghuagai = 1000 × (126.6 × (str2double(a)) − 1870)/((str2double(a))^2 + 51.19 × (str2double(a)) − 476.8);
- % Calculate the amount of activator used:
- bushouji = 1000 × (0.4602 × (str2double(a)) + 3.807)/((str2double(a)) − 9.204);
- chanlv = − 0.0002637 × (str2double(a))^3 + 0.0002637 × (str2double(a))^2 – 0.4094*(str2double(a)) + 65.45;
- % Calculate the concentrate rate.
- pinwei = − 4.3e−005*(str2double(a))^4 + 0.005893*(str2double(a))^3–0.3015 × (str2double(a))^2 + 6.828*(str2double(a)) + 10.96;
- % Calculate concentrate grades:
- huishoulv = 0.0008 × (str2double(a))^3 – 0.09687 × (str2double(a))^2 + 3.705 × (str2double(a)) + 47.08;
- % Calculate the concentrate recovery rate:
- b = num2str(pinwei); % Converts numbers to strings;
- c = num2str(huishoulv); % Converts numbers to strings;
- d = num2str(dianfen); % Converts numbers to strings;
- e = num2str(yanghuagai); % Converts numbers to strings;
- f = num2str(bushouji); % Converts numbers to strings;
- g = num2str(chanlv); % Converts numbers to strings;
- set(handles.edit2, 'string',b) % outputs the string to the text box;
- set(handles.edit3, 'string',c) % outputs the string to the text box;
- set(handles.edit4, 'string', d) % outputs the string to the text box;
- set(handles.edit5, 'string',e) % outputs the string to the text box;
- set(handles.edit6, 'string',f) % outputs the string to the text box;
- set(handles.edit9, 'string',g) % outputs the string to the text box.

Furthermore, according to the above procedures, at the site, the flotation temperature of the flotation circuit can be input in the range from 20 to 45 °C, and the flotation reagent system can be obtained to guide the on-site production practice.

## Results and discussions

### Uniform design factors interaction and optimization

**Figure 3:**
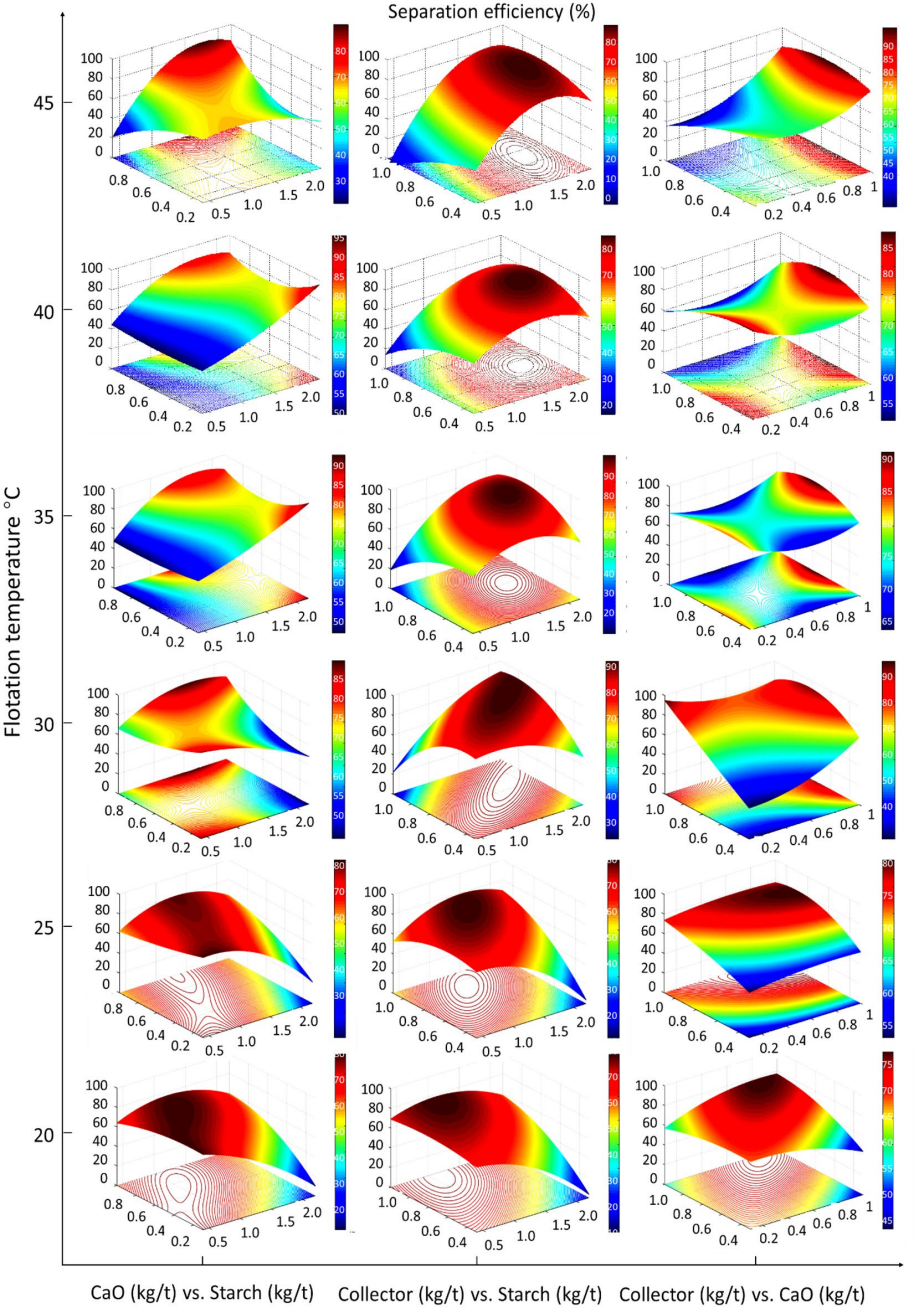
Response surface of flotation reagent system at different flotation temperatures.

The complexity of the flotation process closed-circuit required utilizing a uniform test design to examine the interaction between the independent factors and to find the optimum separation efficiency. Figure 3 shows the response surface modeling of each two factors affecting the separation efficiency while maintaining the other factors at their optimum values at each flotation temperature. CaO is essential for activating quartz particles to accommodate collector adsorption selectively while depressing the iron oxide particles by starch. For instance, at 20 °C, the change in the CaO or collector dosages insignificantly impacted the separation efficiency, especially at lower starch dosage, but increasing the starch dosage required higher dosages of CaO and collector to maintain the high value of separation efficiency. Whereas for the interaction between CaO and collector, the separation efficiency is minimum when one of them is at the highest level and the other at the lowest level, slightly increases when both are at the lowest levels and is maximum when both are at the highest levels. Increasing the flotation temperature enhances the significant effects of these factors. For example, at 45 °C, to accomplish the maximum separation efficiency, the CaO should increase to the maximum value of 1 kg/t with increasing the starch dosage to 1.8 kg/t at an intermediate collector dosage of about 0.7 kg/t. From Fig. 3, the optimum reagent systems giving the maximum separation efficiency (maximum grade and recovery) at each flotation temperature were identified (Table 1). In this study and a previous study^1^ at 30 °C, and at the optimum conditions of 1.6 kg/t depressant dosage, 1.0 kg/t activator dosage, and 0.8 kg/t collector dosage, the maximum Fe grade and recovery of 68.90% and 92.62% respectively were accomplished. In case, the uniform test design is not employed, the typical plant conditions are 1.2 kg/t, 0.5 kg/t, and 0.8 kg/t depressant, activator, and collector respectively; and the plant produces 62.49% concentrate yield containing 68.28% total iron with 89.07% iron recovery^1^.

**Table 1:** Optimum reagent systems at different temperature values.

Flotation temperature (°C)	Flotation reagent system (kg/t)			Yield (%)	Fe grade (%)	Fe recovery (%)
	Starch	CaO	Collector			
20	1.0	0.7	1.2	62.93	67.21	88.83
25	1.25	0.9	1.0	63.14	68.45	91.68
30	1.6	1.0	0.8	63.35	68.90	92.62
35	1.7	1.0	0.8	63.87	68.63	92.42
40	1.8	1.0	0.7	62.88	68.87	91.48
45	1.8	1.0	0.7	62.27	68.40	90.55

### Mathematical models estimation

Given the optimum reagent systems of closed-flotation circuits at different temperature values shown in Table 1, the mathematical models were generated for predicting the concentrate yield, grade, and recovery as well as the optimum flotation reagent systems at different flotation temperatures.

**Figure 4:**
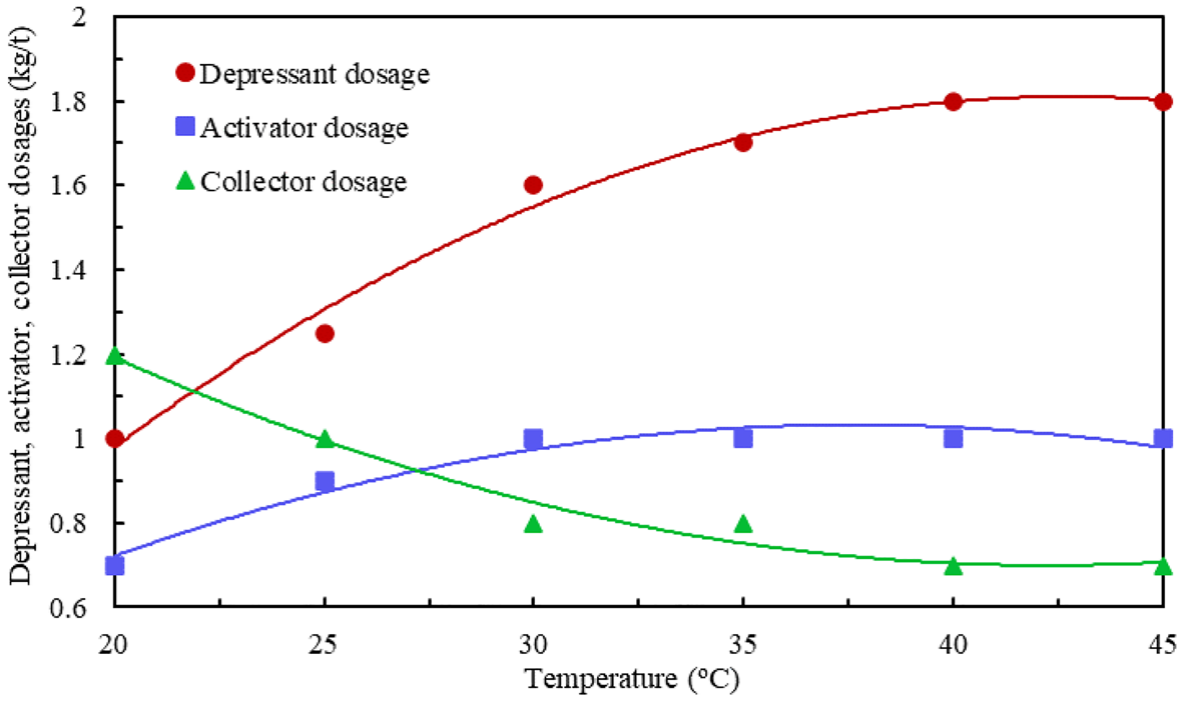
Fitting curves of flotation temperature vs. flotation reagent system.

The fitting curves of the mathematical model of the optimum flotation reagent system (i.e., depressant, activator, and collector dosages) versus the flotation temperature are shown in Fig. 4. It indicates that the increase in flotation temperature from 20 to 45 °C increased the optimum values of both depressant and activator dosages from 1.0 kg/t and 0.7 kg/t to 1.8 kg/t and 1.0 kg/t respectively, but reduced the optimum collector dosage from 1.2 kg/t to 0.7 kg/t.

The mathematical models’ structure of flotation temperature (x) and depressant, activator, and collector dosages (f(x)) are shown in Eqs. (1)–(3) respectively:

1 $$ f\left( x \right) \, = \, \left( {48.1 \times x \, + \, 79.88} \right) \, / \, \left( {x^{2} - \, 57.17 \times x \, + \, 1798} \right) $$

2 $$ f\left( x \right) \, = \, \left( {126.6 \times x \, {-} \, 1870} \right) \, / \, \left( {x^{2} + \, 51.19 \times x \, {-} \, 476.8} \right) $$

3 $$ f\left( x \right) \, = \, \left( {0.4602 \times x \, + \, 3.807} \right) \, / \, \left( {x \, - \, 9.204} \right) $$

The three models fitting effect parameters are relative errors of 0.005022, 0.0004614, and 0.0004639; R^2^ of 0.9907, 0.9937, and 0.976; adjustment R^2^ of 0.9767, 0.9843, and 0.96; and root mean square (RMS) error of 0.05011, 0.01519, and 0.03932 respectively.

**Figure 5:**
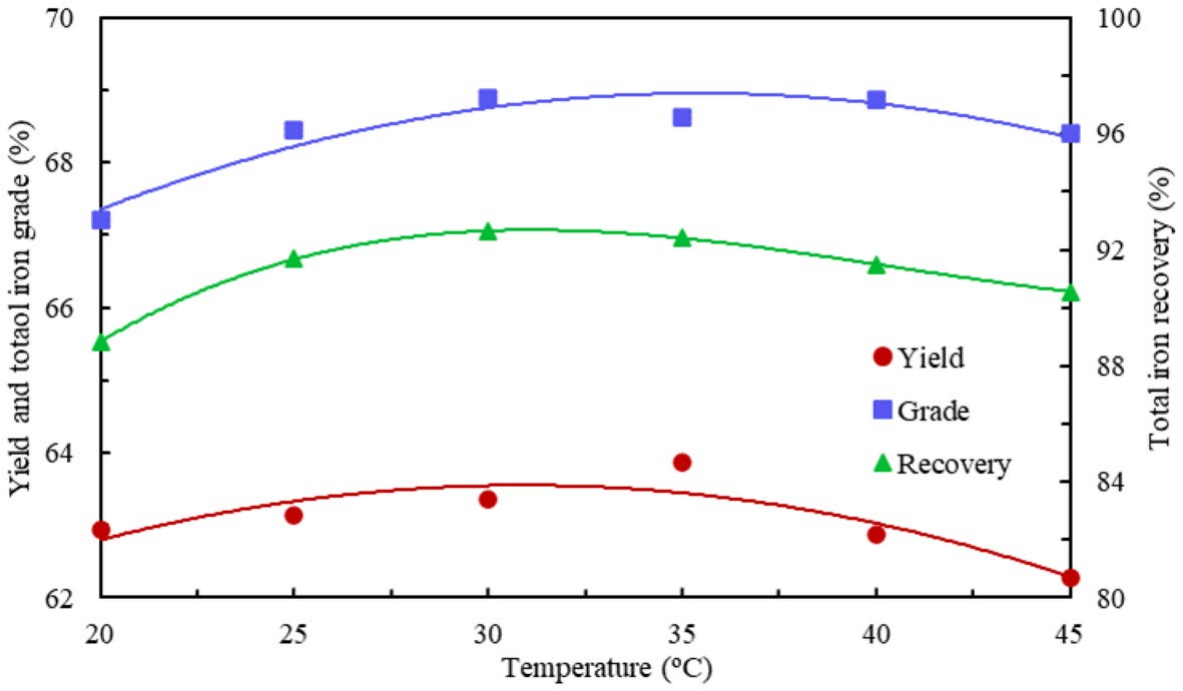
The concentrate yield, grade, and recovery versus flotation temperature at the optimum flotation reagent systems.

At the optimum flotation reagent systems at different flotation temperature conditions, the fitting curves of the mathematical model of concentrate yield, total iron grade, and total iron recovery versus flotation temperature are shown in Fig. 5.

4 $$ f\left( x \right) \, = \, - 0.0002637 \times x^{3} + \, 0.01935 \times x^{2} {-} \, 0.4094 \times x \, + \, 65.45 $$

5 $$ f\left( x \right) \, = \, - \, 4.3 \times 10^{ - 5} \times x^{4} + \, 0.00589 \times x^{3} {-} \, 0.3015 \times x^{2} + \, 6.828 \times x \, + \, 10.96 $$

6 $$ f\left( x \right) \, = \, 0.0008 \times x^{3} - \, 0.09687 \times x^{2} + \, 3.705 \times x \, + \, 47.08 $$

The fitting effect parameters of the three Eqs. (4)–(6) are relative errors of 0.2245, 0.05171, and 0.002071; R^2^ of 0.8418, 0.9734, and 0.9998; adjustment R^2^ of 0.6045, 0.8668, and 0.9995; and RMS error of 0.335, 0.2274, and 0.03218 respectively.

**Figure 6:**
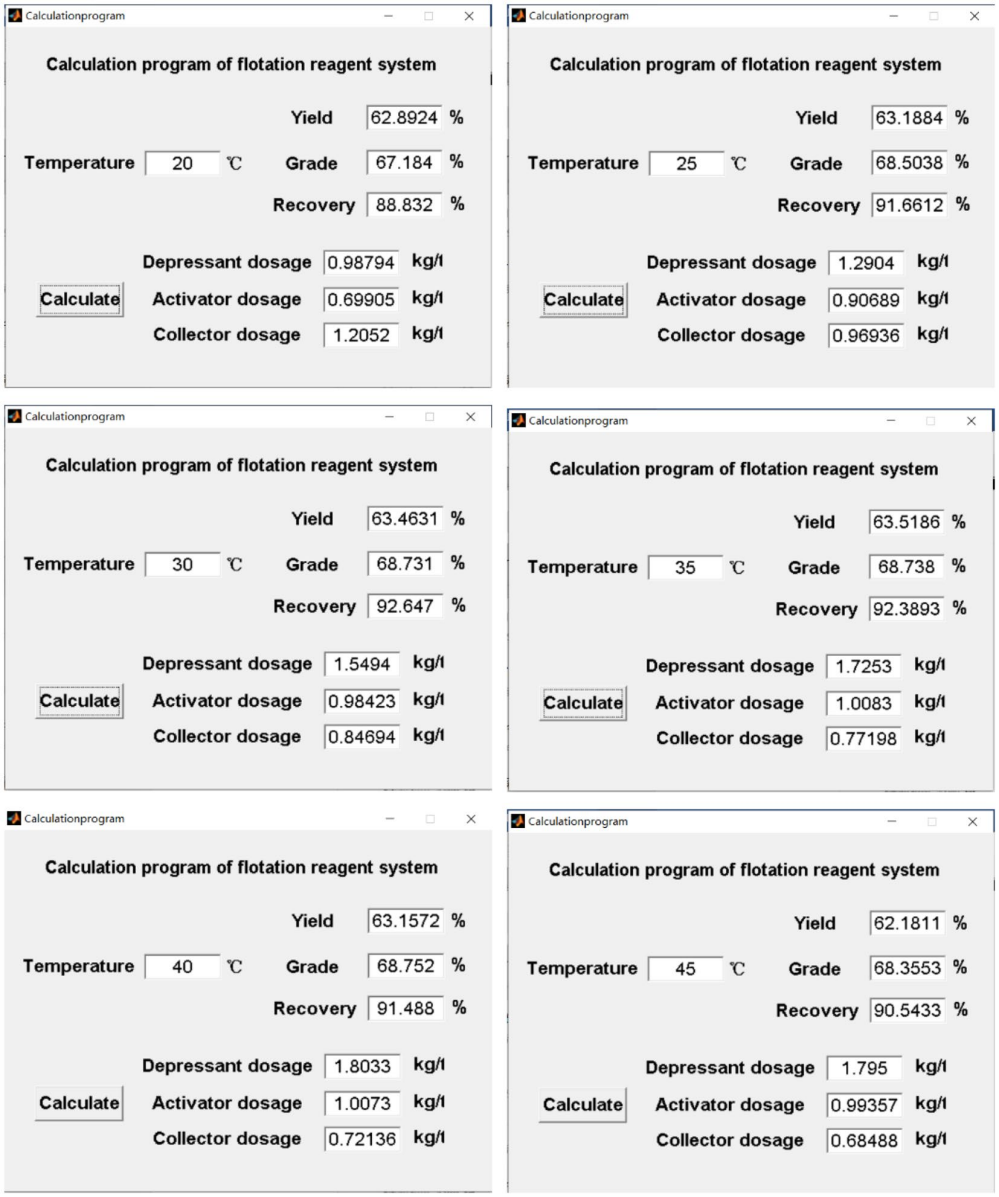
Results of the calculation procedure of the flotation reagent system.

According to the generated models, varying the flotation temperature at the site required adjusting the flotation reagent system with predicting the optimum performance. Thus, the flotation temperature in the flotation circuit can be input in the range from 20 to 45 °C, and the flotation reagent system can be obtained to guide the on-site production practice by giving the expected flotation indexes as shown in Fig. 6.

### Comparative analysis of calculation program and test values

The reagent system obtained by the calculation procedure was compared to the test values under different flotation temperature conditions as shown in Table 2. Also, the flotation results obtained experimentally were compared to the values predicted by the calculation procedure as shown in Table 3, and the relative error values between the test values and the data obtained by the calculation procedure are shown in Table 4. The relative errors are calculated according to Eq. (7)

7 $$ {\text{Relative}}\;{\text{error}} = \, 100x \, \left( {\left| {{\text{Calculated}}\;{\text{ Value}} - {\text{Program}}\;{\text{Value}}} \right|/{\text{Calculated}}\;{\text{Value}}} \right) $$

**Table 2:** Reagent systems obtained by test values and calculation procedures at different temperatures.

Flotation temperature (°C)	Experimental reagent system (kg/t)			Program Calculated reagent system (kg/t)		
	Depressant	Activator	Collector	Depressant	Activator	Collector
20	1.0	0.7	1.2	0.9879	0.6991	1.2052
25	1.25	0.9	1.0	1.2904	0.9069	0.9694
30	1.6	1.0	0.8	1.5494	0.9842	0.8469
35	1.7	1.0	0.8	1.7253	1.0083	0.7720
40	1.8	1.0	0.7	1.8033	1.0073	0.7214
45	1.8	1.0	0.7	1.7950	0.9934	0.6849

**Table 3:** Flotation results by calculation procedure and experimentally under different temperature conditions.

Flotation temperature (°C)	Experimental flotation results			Program calculated flotation results		
	Yield (%)	Grade (%)	Recovery (%)	Yield (%)	Grade (%)	Recovery (%)
20	62.93	67.21	88.83	62.8924	67.1840	88.8320
25	63.14	68.45	91.68	63.1884	68.5038	91.6612
30	63.35	68.90	92.62	63.4631	68.7310	92.6470
35	63.87	68.63	92.42	63.5186	68.7380	92.3893
40	62.88	68.87	91.48	63.1572	68.7520	91.4880
45	62.27	68.40	90.55	62.1811	68.3553	90.5433

**Table 4:** The errors between the test values and calculated values of the closed flotation circuit under different flotation temperature conditions.

Flotation temperature (°C)	The error between the test value and the calculated value (%)			The error between the test value and the calculated value (%)		
	Depressant	Activator	Collector	Yield	Grade	Recovery
20	1.21	0.14	0.43	0.06	0.04	0.00
25	3.23	0.77	3.06	0.08	0.08	0.02
30	3.16	1.58	5.87	0.18	0.25	0.03
35	1.49	0.83	3.50	0.55	0.16	0.03
40	0.18	0.73	3.05	0.44	0.17	0.01
45	0.28	0.64	2.16	0.14	0.07	0.01

It can be seen from Table 4 that the relative error values are small, and the prediction effect of the calculation program is good. Besides, the adjustment of the reagent system can be quickly guided according to the results of the calculation procedure, and the closed-circuit flotation indexes can be obtained.

## Conclusion

In this study, MATLAB-based GUI was utilized to simulate the effect of flotation temperature on the optimum flotation reagent system of the iron oxide reverse flotation circuit. The optimum data were generated first by conducting uniform test design which is a computer simulation utilized in many industrial applications to produce models for presenting real process performance. The increase in flotation temperature from 20 to 45 °C increased the optimum values of both depressant and activator dosages from 1.0 kg/t and 0.7 kg/t to 1.8 kg/t and 1.0 kg/t respectively but reduced the optimum collector dosage from 1.2 to 0.7 kg/t. At these optimum conditions, the product yield, Fe grade, and Fe recovery were in the range of 62.27–63.87%, 67.21–68.90%, and 88.83–92.62% respectively. Then MATLAB-based GUI generated the mathematical models of the optimum reagent systems at different temperatures, so that by varying the flotation temperature at the site, the flotation reagent system can be effectively adjusted with predicting the optimum performance. In summary, GUI is extremely endorsed by mineral processing researchers, and it can also guide the addition of on-site flotation reagents at different flotation temperatures in addition to predicting the flotation indexes such as concentrate yield, grade, and recovery.

## Data Availability

The datasets generated and/or analyzed during the current study are available for the corresponding author upon reasonable request.
